# A Simple Blood Test, Such as Complete Blood Count, Can Predict Calcification Grade of Abdominal Aortic Aneurysm

**DOI:** 10.1155/2017/1370751

**Published:** 2017-08-30

**Authors:** Marika Vezzoli, Stefano Bonardelli, Michele Peroni, Marco Ravanelli, Emirena Garrafa

**Affiliations:** ^1^Department of Molecular and Translational Medicine, University of Brescia, Viale Europa 11, 25123 Brescia, Italy; ^2^Department of Surgery, University of Brescia, Piazzale Spedali Civili 1, 25123 Brescia, Italy; ^3^ASST Spedali Civili di Brescia, Brescia, Italy; ^4^Department of Radiology, University of Brescia, Piazzale Spedali Civili 1, 25123 Brescia, Italy

## Abstract

**Objective:**

The pathogenesis of abdominal aortic aneurysm (AAA) is complex and different factors, including calcification, are linked to increased complications. This study was conducted in order to verify if classical risk factors for AAA and cell blood count parameter could help in the identification of calcification progression of the aneurysm.

**Design:**

Risk factors were collected and cell blood count was performed in patients with AAA and patients were analyzed for the presence of aorta calcification using CT angiography.

**Results:**

We found no association of calcification grade with risk factors for AAA but we found a strong association between MCV, MCH, and calcification grade. Instead, no association was found with the other parameter that we analyzed.

**Conclusions:**

In this study, we demonstrate that biomarkers such as MCV and MCH could have potential important information about AAA calcification progression and could be useful to discriminate between those patients that should undergo a rapid imaging, thus allowing prompt initiation of treatment of suspicious patients that do not need imaging repetition.

## 1. Introduction

Abdominal aortic aneurysm (AAA) is largely an asymptomatic disease, but the aneurysm may rupture with subsequent mortality rates of at least 80% if early detection and elective AAA repair are not performed [1–4]. Most of the literature is devoted to the study of the diameter of AAA since it is known that risk of rupture increases exponentially with maximal aortic diameter, and different authors have reported a relationship with risk factors such as age, smoking history, family history of cardiovascular disease, and dyslipidemia and also with some biomarkers [5–8]. Nevertheless, since aneurysm size does not completely represent the natural history of AAA [5–8] other risk factors, including calcification, have been investigated. Different degrees of mural calcification exist and the gravity of calcification seems to be associated with the risk of rupture [9–11]. Actually no prognostic indices to evaluate progression of calcification exist and repetition of imaging to monitor AAA expansion is necessary, with some important limitations such as cost or availability [12, 13]. Lack of biomarkers for risk stratification of patients with AAA impedes development of novel personalized therapies and interventions since, in every patient with a not-yet “surgical” AAA, there are no clear predictors of a fast or slow progression of its own, AAA; that is, the best interval between a radiological check and the next step is not defined. Different authors have suggested a link between risk factors such as smoking history, obesity, glucose tolerance, dyslipidemia, chronic obstructive pulmonary disease, and renal failure and cardiovascular morbidity and mortality, together with some biomarkers such as RBC indices, WBC counts with differentials, platelet counts and neutrophil-to-lymphocyte ratio (NLR), and platelet-to-lymphocyte ratio (PLR), while no evidence exists in the literature about a possible association between AAA calcification and cell blood count (CBC) parameter even if it is a simple economic and extensively used basic hematological test [14–18]. The aim of our study is to evaluate if classical risk factor and biomarkers associated with AAA [14, 15] can be associated with AAA calcification grade since an accessible and cost-effective measure such as a blood test predicting subsequent AAA progression in calcification could be used to rule in and/or rule out patients for more expensive MR and CT angiography, with benefit for patients and caregivers and with important reduction of cost.

## 2. Methods

### 2.1. Patients

The study enrolled 149 Caucasian patients admitted to the Vascular Surgery of Brescia University “Spedali Civili” hospital in Brescia, Northern Italy, between 2014 and 2016, for AAA surgical repair. Risk factors, including age (continuous), gender (male versus female), and smoking (current versus never or former), were collected. If patients had a body max index > 25 kg/m^2^ they were classified as obese and affected by diabetes mellitus if they had glycated hemoglobin > 6.5% or if they were prescribed antidiabetic drugs. Dyslipidemia was defined as fasting serum low-density lipoprotein cholesterol > 140 mg/dl, triglycerides > 150 mg/dl, or high-density lipoprotein cholesterol < 40 mg/dl or if patients were prescribed lipid-lowering medications. Finally, patients were classified with renal failure when serum creatinine was >2 mg/dl and with chronic obstructive pulmonary disease if they had, during spirometry with a forced expiratory volume in one second, a vital capacity of 70% or less. If cardiovascular disease was present within second-degree relatives, this was recorded as family history of cardiovascular disease. AAA aneurysms were classified on the basis of the anatomical localization and shape. Demographic data and medical history of each patient were collected. Institutional ethic committees approved the study, and all patients provided a written informed consent (approval reference number 1353). Participants did not receive any form of financial compensation. The study conformed to the ethical guidelines of the “World Medical Association Declaration of Helsinki-Ethical Principles for Medical Research Involving Human Subjects” adopted by the 18th World Medical Association General Assembly, Helsinki, Finland, June 1964, and revised in Tokyo in 2004.

### 2.2. Imaging Assessment of Aneurysm Calcifications

Aneurysm calcifications were qualitatively assessed by the consensus of two physicians: a radiologist and a resident in vascular surgery based on the CT angiography performed within one month before surgery. By a single radiologist, calcifications were evaluated on axial multiplanar reconstructions using a 10 mm thick maximum intensity projection on three different levels: upper, middle, and lower portion of the aneurysm. Calcification grade was scored as I when calcifications covered less than one-third of aortic circumference and as II when they covered more than one-third of aortic circumferences. The score at the upper and lower aneurysm level was multiplied by a factor of 0.5 in order to reflect the changes in the aneurysm circumference due to the aneurysm shape. A global score I was observed in 88 patients and a score II in 61 patients.

### 2.3. Blood Collection and Laboratory Measurements

CBC information used in this analysis was from blood samples drawn from fasting overnight patients via an antecubital vein puncture before AAA resection and commercially available assays were used according to manufacturer's instruction. Specimens were collected in peripheral blood sampling microtainer tube containing K_2_EDTA and complete blood count was measured with the Coulter LH 750 automatic blood counting system. Red blood cell (RBC) indices (hemoglobin, Hb; mean corpuscular volume, MCV; mean corpuscular hemoglobin, MCH; mean corpuscular hemoglobin concentration, MCHC; and red blood cell distribution width, RDW), white blood cell (WBC) counts with differentials (neutrophil; lymphocyte; monocyte, eosinophils, and basophils) and platelet (PLT) counts data were collected. NLR was calculated by dividing absolute neutrophil count by absolute lymphocyte count and PLR as the ratio of the platelet to lymphocyte. The instruments were calibrated against appropriate proprietary reference standard material and verified by using the registered quality controls.

### 2.4. Statistical Analysis

To analyze the relationships between calcification and the variables in the dataset, we applied different test.

First of all, we tested the association between calcification and the 9 risk factors in the dataset (which are qualitative variables) using Pearson's Chi-squared test.

For the 14 quantitative variables related to blood count, we studied possible relationships with calcification by means of the nonparametric Wilcoxon signed-rank test since all the variables (except one) are not normally distributed. Wilcoxon signed-rank test is a good alternative to *t*-test when the population cannot be assumed to be normally distributed. Moreover, for the variables related to calcification, we build a boxplot in order to clearly highlight the differences between patients with severe calcifications and the others.

These procedures were performed with the statistical programming language R version 3.2.4.

## 3. Results

The dataset is composed of 149 observations, 13 females (8.72%) and 136 males (91.28%). Among them, 88 patients (59.06%) showed a calcification that covered less than 33% of aortic circumference while for the remaining 61 the calcification covered more than 33% of aortic circumferences. Patients differed in age, sex, hypertension, obesity, glucose tolerance, renal failure, family history of cardiovascular diseases, and chronic obstructive pulmonary disease. As shown in Table 1, in our data no significant association was found with the classical risk factors analyzed between patients with calcification grades I and II.

We then analyzed CBC and, overall, all hematological indices were within the normal limit according to our laboratory references.  Table 2 reports the descriptive statistics for the 14 quantitative variables in the dataset related to blood count.

We note that, for neutrophils, lymphocytes, monocytes, eosinophils, and basophils, the Interquartile Range (IR, henceforth) is equal to 0 (there is no variability in the central interval that contains 50% of the ordered observations).

Table 3 reports the results of Shapiro-Wilk normality test, pointing out that all the variables (except RBC) are not normally distributed. These results led us to use nonparametric Wilcoxon signed-rank test since it does not require particular assumptions on the distribution of the analyzed variables. In fact, on this type of data,* t*-test could lead to biased results. Wilcoxon *p* values are reported in Table 4.

For completeness, we compute also *t*-test for the unique variable normally distributed RBC which provides similar results (see Table 5) to the Wilcoxon signed-rank test. We computed also the following ratios well known in literature: NLR and PLR. For both we reject the hypothesis of normality (see Table 3) and we use the Wilcoxon signed-rank test for understanding potential relationships with wall calcification (Table 4).

**Figure 1:**
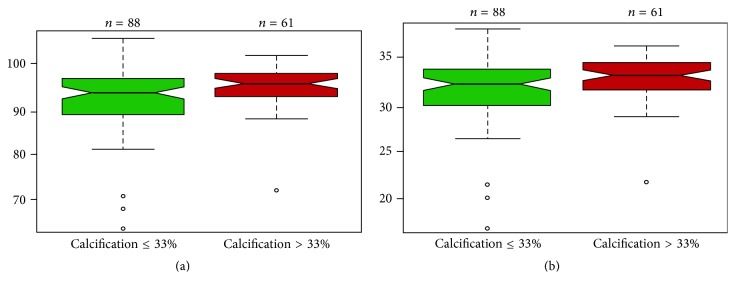
Boxplots for MCV (a) and MCH (b) using grouping variable calcification.

Only MCV and MCH (Table 4) show a significant relationship with calcification (*p* value < 0.05). More precisely, patients with severe calcification (>33%) have higher median values for both variables (see also red boxplots in Figures 1(a) and 1(b)). Since reference values for MCV are different for men and women, we repeated the Wilcoxon signed-rank test excluding the 13 females from the analysis. It is interesting to note that we obtained similar results (see Table 6).

Differently from other studies related to calcification and other cardiovascular disease, no significant difference was observed in all the other parameters observed.

## 4. Discussion

The main finding in our study is the identification of potential biomarkers of increased risk of calcification in patients with AAA. Our results, if confirmed in independent larger studies, may have potential implications for improved prediction, therapy personalization, and development of novel therapies. Personalized medicine is the concept promising progress in modern healthcare, and the biomarkers comprise its cornerstone [19]. Despite obviously varying rates of calcification progression or further clinical destabilization, current guidelines recommend a universal approach to these “high risk” patients. Such uniform management may be responsible for lack of progress in development of new strategies in the management of these patients. In our population of 149 patients admitted to the vascular surgery for AAA restriction we found that well-known risk factors for AAA are not, in our hands, correlated with different grade of calcification suggesting that none of the risk factors analyzed can be used reliably as risk factors for progression of calcification.

As an economic and extensively used basic hematological test, some parameter of CBC became a target of investigation after it was found that some of them are associated with morbidity, mortality, and calcification in different cardiovascular diseases [16, 20, 21].

A large number of studies use* t*-test to determine if two sets of data are significantly different from each other, without checking the normality in the units. For this reason, we first control the data distribution and we then choose a tailored approach able to deal with observations non-normally distributed. Consequently, we are confident that our results are reliable.

In our analysis we highlight a statistically significant difference in median values of MCV and MCH (both *p* values are lower than 0.05) in patients with different grade of calcification of AAA, confirming that these subgroups of individuals come from different populations.

Reduction in RBC indices, such as MCV and MCH, accompanies the aging of RBC together with a decrease in whole cell deformability while rigidity increases this reduction in deformability which plays a role in the destruction of the RBC [22–24]. Deformability describes the ability of RBCs to change shape in response to deforming forces which not only improves their flow properties but also protects against cell disruption under bulk flow conditions and in the circulation when passing through vessels with higher rigidity caused, for example, by calcification, or with a flow shear stress caused by the change in the structure of the calcified AAA [25, 26]. Probably the reason of the higher MCV and MCH in patient with a higher calcification grade is related to the fact that older RBCs, with lower MCV and lower MCH, that have less elasticity died easily following turbulence that can be present nearby AAA calcification or cannot support the impact with calcified tissue that has as a consequence a global MCV and MCH increase.

From our data we found no association of the other above-mentioned markers with different grade of calcification of AAA; this is probably dependent on the fact that markers described to correlate with calcification of other districts are not useful for AAA calcification grade, regarding the heterogeneity between previous studies and our studies in the studied population and definition of each calcified group as well as imaging modalities to detect calcification, and from a methodological point of view statistical analysis used by most of the other studies was less complex than the one we use in our study. Nevertheless, our findings suggest that none of CBC indices can be used reliably as a marker of calcification grade of AAA apart from MCV and MCH that strongly correlate with the different grade of calcification. This is very important since, beside imaging, today no accurate methods exist in order to diagnose calcified AAAs, and clinical examination is still doubtful and the management of patients with AAA might significantly benefit from the measurement of circulating markers that facilitate an early diagnosis and that could have a direct correlation with a possible fast growth of a known lesion with important limitation such as cost, availability, or waiting time; therefore the aim of our study is to identify circulating markers that could substantially help to identify appropriate patients for different monitoring protocols and intervention. We also believe that this marker should be available in most of the laboratories and have a weak economic impact.

## Figures and Tables

**Table 1:** 

Variables	*p* value
Smoking history	0.1088
Obesity	1.0000
Glucose tolerance	0.9164
Dislipidemia	0.7970
Family history cardiovascular disease	0.1520
Chronic obstructive pulmonary disease	0.7771
Renal failure	0.5304
Localization	0.5078

*p *values were computed using Pearson's Chi-squared test.

**Table 2:** Descriptive statistics for variables related to blood count.

Statistics	WBC	RBC	Hgb	Hct	MCV	MCH	MCHC	RDW	PTL	Neutrophils	Lymphocytes	Monocytes	Eosinophils	Basophils
Mean	7.17	4.40	13.79	43.48	93.72	31.49	33.39	14.53	192.82	4.33	1.75	0.58	0.32	0.03
Error standard	0.15	0.05	0.14	2.45	0.50	0.19	0.21	0.12	4.52	0.09	0.04	0.01	0.15	0.00
Mode	5.73	4.74	15.20	44.20	93.80	31.40	33.30	13.70	180.00	4.18	1.72	0.56	0.15	0.03
Q1	5.89	4.06	12.8	38.3	91.6	30.5	33.1	13.6	152	4.18	1.72	0.56	0.15	0.03
Q2 (median)	7.01	4.48	14.00	42.00	94.10	31.70	33.60	14.20	188.00	4.18	1.72	0.56	0.15	0.03
Q3	8.34	4.78	14.90	44.20	97.10	32.90	34.10	15.20	218.00	4.18	1.72	0.56	0.15	0.03
IR = Q3 − Q1	1.33	0.30	0.90	2.20	3.00	1.20	0.50	1.00	30.00	0.00	0.00	0.00	0.00	0.00
Min	3.87	2.55	8.20	24.30	65.10	20.20	3.70	12.20	11.00	1.96	0.70	0.34	0.02	0.00
Max	12.52	6.29	17.30	401.00	106.90	36.80	35.50	19.40	387.00	9.52	3.52	1.12	22.00	0.09
Range (max − min)	8.65	3.74	9.10	376.70	41.80	16.60	31.80	7.20	376.00	7.56	2.82	0.78	21.98	0.09
Standard deviation	1.80	0.60	1.65	29.91	6.06	2.32	2.56	1.43	55.18	1.06	0.45	0.12	1.79	0.01
Sample variance	3.25	0.36	2.74	894.33	36.75	5.38	6.54	2.06	3044.35	1.13	0.21	0.01	3.21	0.00
Kurtosis	−0.13	0.77	0.65	140.68	5.49	5.33	125.00	2.00	1.47	6.75	3.68	4.69	148.24	6.39
Asymmetry	0.48	−0.28	−0.63	11.69	−1.55	−1.49	−10.71	1.36	0.62	1.84	1.09	1.61	12.16	1.55
Coefficient of variation	0.25	0.14	0.12	0.69	0.06	0.07	0.08	0.10	0.29	0.25	0.26	0.21	5.63	0.40

**Table 3:** Results of the Shapiro-Wilk normality on the variables related to the blood count.

Shapiro test	
Variables	*p* value
WBC	0.0145
RBC	0.0844
Hgb	0.0026
Hct	<2.2*E* − 16
MCV	4.36*E* − 09
MCH	1.56*E* − 08
MCHC	<2.2*E* − 16
RDW	3.90*E* − 09
PTL	0.0005
Neutrophils	5.88*E* − 15
Lymphocytes	2.75*E* − 13
Monocytes	2.56*E* − 14
Eosinophils	<2.2*E* − 16
Basophils	2.96*E* − 16
NLR	<2.2*E* − 16
PLR	2.23*E* − 11
MLR	<2.2*E* − 16
ELR	<2.2*E* − 16
BLR	2.20*E* − 16

**Table 4:** Wilcoxon signed-rank test for variables related to blood count. In bold,* p *value* < *0.05.

Variables	Median	*p* value	Min–max	95th perc
WBC				
Calcification ≤ 33%	7.06	0.8379	3.87–12.52	10.42
Calcification > 33%	6.79		4.20–11.83	10.54
RBC				
Calcification ≤ 33%	4.58	0.1488	2.55–6.29	5.30
Calcification > 33%	4.37		2.89–5.40	5.14
Hgb				
Calcification ≤ 33%	14.10	0.9047	8.20–17.10	16.03
Calcification > 33%	14.00		9.40–17.30	16.00
Hct				
Calcification ≤ 33%	42.45	0.9784	24.30–51.10	47.36
Calcification > 33%	41.80		27.00–401.00	49.30
MCV				
Calcification ≤ 33%	93.70	**0.0172**	65.10–105.20	101.43
Calcification > 33%	95.60		73.20–106.90	101.10
MCH				
Calcification ≤ 33%	31.40	**0.0168**	20.20–35.70	34.13
Calcification > 33%	32.10		23.80–36.80	34.40
MCHC				
Calcification ≤ 33%	33.50	0.1402	3.70–35.30	34.40
Calcification > 33%	33.80		32.20–35.50	34.90
RDW				
Calcification ≤ 33%	14.20	0.4062	12.20–19.40	17.33
Calcification > 33%	14.10		12.80–19.00	16.90
PTL				
Calcification ≤ 33%	189.00	0.6824	11.00–387.00	294.55
Calcification > 33%	188.00		99.00–354.00	265.00
Neutrophils				
Calcification ≤ 33%	4.18	0.4997	1.96–9.52	6.36
Calcification > 33%	4.18		2.04–7.78	6.39
Lymphocytes				
Calcification ≤ 33%	1.72	0.4597	0.75–3.52	2.75
Calcification > 33%	1.72		0.70–3.16	2.24
Monocytes				
Calcification ≤ 33%	0.56	0.6664	0.34–1.12	0.84
Calcification > 33%	0.56		0.34–1.03	0.84
Eosinophils				
Calcification ≤ 33%	0.15	0.2171	0.02–22.00	0.39
Calcification > 33%	0.15		0.02–0.64	0.37
Basophils				
Calcification ≤ 33%	0.03	0.1931	0.00–0.09	0.06
Calcification > 33%	0.03		0.00–0.06	0.04
NLR				
Calcification ≤ 33%	2.43	0.9814	0.80–12.69	4.27
Calcification > 33%	2.43		1.19–11.00	4.25
PLR				
Calcification ≤ 33%	107.56	0.7929	4.72–386.67	225.71
Calcification > 33%	109.88		58.23–250.54	205.81
MLR				
Calcification ≤ 33%	0.33	0.8441	0.15–1.13	0.52
Calcification > 33%	0.33		0.20–1.29	0.56
ELR				
Calcification ≤ 33%	0.09	0.1405	0.02–14.77	0.23
Calcification > 33%	0.09		0.02–0.36	0.18
BLR				
Calcification ≤ 33%	0.02	0.5956	0.00–0.09	0.03
Calcification > 33%	0.02		0.00–0.04	0.03

In bold, *p* values < 0.05.

**Table 5:** *t*-test for RBC variable.

Variable	Mean	*p *value	Min–max	95th perc
RBC				
Calcification ≤ 33%	4.45	0.1981	2.55–6.29	5.30
Calcification > 33%	4.32		2.89–5.40	5.14

**Table 6:** Wilcoxon signed-rank test for MCV and MCH excluding females from the analysis.

Variables	Median	*p* value	Min–max	95th perc
MCV				
Calcification ≤ 33%	93.10	**0.0060**	65.10–105.20	101.50
Calcification > 33%	95.70		73.20–106.90	101.15

In bold, *p* values < 0.05.
